# Irisin Contributes to Neuroprotection by Promoting Mitochondrial Biogenesis After Experimental Subarachnoid Hemorrhage

**DOI:** 10.3389/fnagi.2021.640215

**Published:** 2021-02-03

**Authors:** Tianqi Tu, Shigang Yin, Jinwei Pang, Xianhui Zhang, Lifang Zhang, Yuxuan Zhang, Yuke Xie, Kecheng Guo, Ligang Chen, Jianhua Peng, Yong Jiang

**Affiliations:** ^1^Department of Neurosurgery, The Affiliated Hospital of Southwest Medical University, Luzhou, China; ^2^Luzhou Key Laboratory of Neurological Diseases and Brain Function, The Affiliated Hospital of Southwest Medical University, Luzhou, China; ^3^Academician (Expert) Workstation of Sichuan Province, The Affiliated Hospital of Southwest Medical University, Luzhou, China; ^4^Sichuan Clinical Research Center for Neurosurgery, The Affiliated Hospital of Southwest Medical University, Luzhou, China

**Keywords:** FNDC5/irisin, subarachnoid hemorrhage, mitochondrial homeostasis, oxidative stress, neuronal apoptosis

## Abstract

Subarachnoid hemorrhage (SAH) is a devastating form of stroke, which poses a series of intractable challenges to clinical practice. Imbalance of mitochondrial homeostasis has been thought to be the crucial pathomechanism in early brain injury (EBI) cascade after SAH. Irisin, a protein related to metabolism and mitochondrial homeostasis, has been reported to play pivotal roles in post-stroke neuroprotection. However, whether this myokine can exert neuroprotection effects after SAH remains unknown. In the present study, we explored the protective effects of irisin and the underlying mechanisms related to mitochondrial biogenesis in a SAH animal model. Endovascular perforation was used to induce SAH, and recombinant irisin was administered intracerebroventricularly. Neurobehavioral assessments, TdT-UTP nick end labeling (TUNEL) staining, dihydroethidium (DHE) staining, immunofluorescence, western blot, and transmission electron microscopy (TEM) were performed for post-SAH assessments. We demonstrated that irisin treatment improved neurobehavioral scores, reduced neuronal apoptosis, and alleviated oxidative stress in EBI after SAH. More importantly, the administration of exogenous irisin conserved the mitochondrial morphology and promoted mitochondrial biogenesis. The protective effects of irisin were partially reversed by the mitochondrial uncoupling protein-2 (UCP-2) inhibitor. Taken together, irisin may have neuroprotective effects against SAH *via* improving the mitochondrial biogenesis, at least in part, through UCP-2 related targets.

## Introduction

Subarachnoid hemorrhage (SAH) accounts for 5–10% in all stroke events (Lawton and Vates, 2017). With extremely high mortality (nearly 50%) and disability rate (over 30%), this form of stroke causes a severe health problem worldwide. For decades, studies have focused on the processes of early brain injury (EBI) after SAH, which was widely attributed to be one of the primary causes of the poor outcomes (Cahill et al., 2006; Cahill and Zhang, 2009). Recently, mitochondrial homeostasis during the EBI process has gradually become the focus of studies (Hagberg et al., 2014).

Mitochondria, as the core of energy metabolism, are strongly associated with the homeostasis of cell metabolism. Previous studies have emphasized on the necessity of improving mitochondrial dysregulation when it comes to neuroprotection (Hagberg et al., 2014). The brain is an important organ with high metabolic rates, and the imbalance of energy metabolism, which is caused by the dysfunction of mitochondria after brain injury, would lead to pathological cascades in EBI (Sims and Muyderman, 2010; Hayakawa et al., 2016). Considering the significant role of mitochondria, the exploration of innovative mitochondria-targeted therapeutic strategies of brain injury is prospective (Bolanos et al., 2009).

Irisin is a cleaved version of fibronectin domain-containing protein 5 (FNDC5), a membrane protein comprising a short cytoplasmic domain (Lourenco et al., 2019). Recently, the actual existence of irisin has been verified in myocytes, plasma, and brain tissues (Dun et al., 2013; Piya et al., 2014; Ruan et al., 2018). Meanwhile, the outstanding effects of irisin in metabolism regulation, especially in lipid metabolism and mitochondrial homeostasis, make this cytokine to attract much attention in the research field (Bostrom et al., 2012; Hocking et al., 2013; Farmer, 2019). More importantly, irisin has been proved to be an effective intervention for reducing damage, promoting recovery, and improving prognosis in acute brain injury studies (Li et al., 2017; Tu et al., 2020).

In the current study, we focused on the role of irisin in neuroprotective effects and the underlying mitochondria-related mechanisms, shedding light on the influence of irisin after SAH.

## Materials and Methods

### Animals

All experimental procedures were approved by the Care and Use of Laboratory Animals of China and were following the guidelines of the Animal Committee of the Ethics Committee of Southwest Medical University. Male C57BL/6J mice (aged 8–12 weeks with an average weight of 18–22 g) were obtained from Chengdu Dashuo Experimental Animal Co., Ltd. All mice were housed in a room with a 12-h light/dark cycle and with controlled humidity and temperature. All of the animals could freely access fresh food and clean water.

### SAH Model

The endovascular perforation model of SAH was performed as previously described (Peng et al., 2019b). Briefly, mice were inductively anesthetized in a hermetic box with 3–4% isoflurane, and then mice were masked and ventilated with 1–1.5% isoflurane throughout the operation. In a supine position, the skin of the neck was opened with a sharp scalpel in the midline. Then, the common carotid artery (CCA), external carotid artery (ECA), and internal carotid artery (ICA) of the right side were exposed. A 5.0 filament was inserted into the right ICA through the isolated right ECA. After a resistance was felt at the bifurcation of the anterior and middle cerebral arteries, the suture was advanced 2 mm further to perforate the vessel during immediate withdrawal. We monitored the occurrence of the typical Cushing response as a secondary judgment of the success of the SAH model. The mice in the sham group underwent the same procedures without vessel perforation. After the filament was removed and the stump of the ICA was ligated, the skin incision was sutured and disinfected with diluted iodophor. The mice were monitored at 25°C with warm sets of cage every 15 min until they recovered from anesthesia, and then they were transferred back to their home cages.

### SAH Grading and Mortality Analysis

The assessment of the SAH grading score was performed by two independent investigators blinded to the experimental design at 24 h after SAH, as previously described (Wu Y. et al., 2019). The basal cistern of the mouse brain was divided into six regions, and according to the amount of blood clotting, each region was assigned a grade ranging from 0 to 3. The total score for the six regions was defined as the mouse SAH grade. Briefly, total grades of 0–7, 8–12, and 13–18 indicated mild, moderate, and severe SAH, respectively. In this study, mice with the SAH grade < 8 were excluded. Mortality was calculated as the number of dead mice divided by the total number of mice used after SAH in the experiment.

### Study Design

Four experiments were conducted as follows.

#### Experiment 1

Typical time points in the EBI process were chosen to detect the temporal expression of endogenous irisin within the ipsilateral hemisphere of the brain after SAH. A total of 36 mice were randomly divided into six groups for Western blotting: Sham (*n* = 6), SAH-6 h (*n* = 6), SAH-12 h (*n* = 6), SAH-24 h (*n* = 6), SAH-48 h (*n* = 6), and SAH-72 h (*n* = 6). Additionally, six mice in the Sham (*n* = 3) and the SAH-24 h (*n* = 3) group were used for FNDC5/irisin spatial co-localization *via* immunofluorescence.

#### Experiment 2

To optimize the concentration of exogenous irisin used in the experiments, three concentration gradients of exogenous irisin were designed, and 30 mice were divided into five groups: Sham (*n* = 6), SAH+vehicle (0.9% NaCl, *n* = 6), SAH+irisin (150 μg/kg, *n* = 6), SAH+irisin (300 μg/kg, *n* = 6), and SAH+irisin (600 μg/kg, *n* = 6). Neurological scores and SAH grades were evaluated 24 h after SAH. To reduce any subjective bias, all mice were marked with a marker pen on their tails by an experimenter to distinguish between groups, and another two investigators who were blinded to the groups' information performed the tests. Based on the results of the above tests, irisin-treated mice (300 μg/kg) were used for future experiments.

#### Experiment 3

A total of 36 mice were divided into three groups randomly: Sham (*n* = 12), SAH+vehicle (0.9% NaCl *n* = 12), and SAH+irisin (300 μg/kg, *n* = 12). Western blotting, immunofluorescence, TdT-UTP nick end labeling (TUNEL) staining, dihydroethidium (DHE) staining, and assay kits were implemented to evaluate neuronal apoptosis and oxidative stress at 24 h after SAH. Meanwhile, transmission electron microscopy (TEM) was performed to measure the change of the number, morphology, and function of mitochondria. Western blotting and immunofluorescence were also used to analyze the expression of proteins related to mitochondrial biogenesis at 24 h after SAH.

#### Experiment 4

To explore the mechanism of the neuroprotective role of irisin, a total of 36 mice were randomly assigned into four groups: Sham (*n* = 9), SAH+vehicle (0.9% NaCl, *n* = 9), SAH+irisin+vehicle (300 μg/kg-irisin, 0.9% NaCl, *n* = 9), and SAH+irisin+Genipin (300 μg/kg-irisin, 30 mg/kg-Genipin, *n* = 9). Western blotting, TUNEL staining, and immunofluorescence were used to analyze the effects of genipin, the inhibitor of uncoupling protein-2 (UCP-2), on the protective roles of exogenous irisin at 24 h after SAH. Additionally, to estimate the influence of genipin, four groups were added: naive+vehicle, naive+genipin, SAH+vehicle, and SAH+genipin.

### Neurological Performance Evaluation

Two independent investigators who were blinded to the experimental design information evaluated the neurological performance to avoid any bias. The modified Garcia scale and the beam balance tests were used to evaluate neurological scores as previously described (Xie et al., 2020). The modified Garcia scale (maximum score = 18) included the tests of spontaneous activity (0–3), the spontaneous movement of the four limbs (0–3), forelimbs outstretching (0–3), climbing capacity (1–3), response to vibrissae touch (1–3), and trunk touch (1–3). The ability of mice to walk on a round wooden beam within 1 min was evaluated using the beam balance test (0–4), and the mean score was calculated based on three consecutive trials scored from 0 to 4 according to the walking ability.

### Drug Administration

Intracerebroventricular administration was performed as previously described (Xie et al., 2020). Briefly, after mice were anesthetized, the scalps were opened through the midline, and a burr hole was drilled with a needle of a 50 ml syringe (0.3 mm anterior and 1 mm lateral to the bregma). Then, the needle of a 10-μl microsyringe was inserted into the hole (3 mm in depth) to enter the lateral ventricle. Exogenous irisin diluted in a normal saline solution (067-29A, Phoenix Pharmaceuticals, USA) was injected intracerebroventricularly 30 min after SAH induction. Similarly, the normal saline (vehicle), which does not contain irisin, was injected into the vehicle groups as controls.

To explore whether the effects of irisin is associated with the upregulation of UCP-2, genipin, a specific UCP-2 inhibitor, was injected intravenously before the administration of irisin in experimental animals with SAH. Briefly, at 30 min before SAH, the animals were injected with genipin diluted in a normal saline solution (30 mg/kg body weight; Solarbio, China) *via* the caudal vein. After 30 min of SAH, irisin administration was performed as previously described. Similarly, the animals in the vehicle group were treated intravenously with an equivalent amount of the normal saline solution without genipin 30 min before SAH, and then, an equivalent amount of the normal saline solution without irisin was treated intracerebroventricularly 30 min after SAH.

### Immunofluorescence Staining

Double fluorescence staining was performed as previously described (Pang et al., 2018). The slices were rewarmed at room temperature for 10 min and permeabilized with 0.1% Triton X-100 (diluted in PBS) for 5 min. After being washed with PBS three times (5 min per time), the slices were blocked with 10% goat serum (diluted in PBS) for 2 h at room temperature. Then, the slices were incubated overnight (about 12 h) at 4°C with primary antibodies, including anti-irisin (ab131390, Abcam, USA, 1:100) and anti-NeuN (ab104224, Abcam, USA, 1:100).

Next, the slices were washed with PBS and incubated with the appropriate secondary antibody (1:200) at room temperature for 2 h. Finally, 4',6-diamidino-2-phenylindole (DAPI; C0060, Solarbio, China, 1:200) were used to stain the cell nucleus for 5 min at room temperature. After dropping the anti-fluorescence attenuation agents (20 μl per tissue) and covering the cover slides, the slices were observed and recorded with a fluorescence microscope. Next, three random coronal sections per brain were selected, and the defined regions around the puncture point were observed in the right hemisphere for analysis. Photographs and fluorescence intensity were analyzed with Image-Pro Plus 6.0 software (MediaCybernetics, USA).

### DHE Staining

To assess the oxidative stress level in the brain, double staining of neurons and DHE (D7008, Sigma-Aldrich, USA) was used to stain the frozen brain slices at 24 h after SAH. Neurons were stained by a neuron marker, the neuronal nuclear protein (NeuN; ab104224, Abcam, USA, 1:100). Freshly prepared slices were incubated with 3 μmol/l DHE in a humidified chamber and protected from light (at 37°C incubator for 45 min). Photographs and the fluorescence intensity were analyzed with Image Image-Pro Plus 6.0 software (MediaCybernetics, USA).

### TUNEL Staining

To quantify the neuronal apoptosis level, double staining of neurons and TUNEL-positive cells was performed at 24 h after SAH. Neurons were stained by the neuron marker NeuN (ab104224, Abcam, USA, 1:100), and TUNEL staining was done according to the manufacturer's instructions of *in situ* Cell Death Detection Kit, TMR red (Roche, USA). Three microscopic fields of each slice were observed and recorded by a fluorescence microscope, and the TUNEL-positive neurons around the puncture point were counted. Data were presented as the apoptotic index, TUNEL-positive neurons/total neurons in %.

### Western Blotting

Western blotting analysis was performed as previously described (Peng et al., 2019a). The total proteins were extracted from the right brain hemisphere tissues, and bicinchoninic acid (BCA) assay kits (Beyotime, China) were used to determine protein concentration. Equal amounts of a sample protein were loaded onto an SDS-PAGE gel for protein separation. Then, samples were transferred onto a nitrocellulose membrane (0.20 μm, Millipore) by using a wet transfer system. After the membrane transfer was finished, the membrane was blocked in 5% non-fat dry milk (diluted in Tris-Buffered Saline and Tween 20, TBST) for 2 h at room temperature. Next, the membrane was washed three times with TBST (5 min per time) and incubated with the following primary antibodies at 4°C overnight: anti-irisin (ab131390, Abcam, USA, 1:1,000), anti-TFAM (ab131607, Cell Signaling Technology, USA, 1:1,000), anti-PGC-1α (ab2178s, Cell Signaling Technology, USA, 1:1,000), anti-Cleaved caspase-3 (9661, Cell Signaling Technology, USA, 1:1,000), anti-Bcl-2 (1:1,000, Proteintech, China, 1:1,000), anti-Bcl-2-associated X protein (Bax; 1:1,000, Cell Signaling Technology, USA, 1:1500), anti-SOD-2 (24127-1-AP, Proteintech, China, 1:2,000), anti-UCP-2 (11081-1-AP, Proteintech, China, 1:1500), and anti-β-actin (20536-1-AP, Proteintech, China, 1:6,000).

Then, the membrane was washed five times with TBST (5 min per time), and the secondary antibody of SA00001-2 (Proteintech, China, 1:6,000) was incubated for 1 h at room temperature. The membranes were washed five times with TBST (5 min per time) before the immunoblots were taken for visualization. The bands were displayed by using enhanced electrogenerated chemiluminescence (ECL; Fdbio Science) and photographed by the ChemiDoc Imaging System (Bio-Rad, Hercules, USA). Immunoblot band images were analyzed by Image Pro-Plus 6.0 software (MediaCybernetics, USA).

### Assay Kits of Oxidative Stress

For detecting the change of the levels of oxidative stress in mice brain at 24 h after SAH, fresh brain tissues were collected, and samples were prepared as per the manufacturer's instructions. Superoxide dismutase (SOD) Assay Kit (S0103, Beyotime, China), Malonaldehyde (MDA) Assay Kit (S0131, Beyotime, China), and Glutathione Peroxidase Activity (GSH-PX) Assay Kit (S0052, Beyotime, China) were used as the instructions illustrated.

### Transmission Electron Microscopy

Transmission electron microscopy was performed as previously reported (Gao et al., 2017). Briefly, under deep anesthesia, the mice were perfused transcardially with pre-cooled PBS (40 ml per mouse). Then, the right brain tissues around the puncture point in the SAH groups and their corresponding areas in the sham group of the mice were collected and fixed in 2.5% glutaraldehyde (diluted in PBS) at 4°C overnight. After the tissues were washed four times with PBS (5 min per time) and stayed for 15 min, these brain tissues were fixed in 1% Osmium acid (diluted in PBS) at 4°C for 2 h. Next, the brain tissues were dehydrated in different concentrations of ethanol. Then, these samples were buried into the embedding medium and cut into 70-nm-thick slices by using an ultramicrotome (EM UC7, Leica, Germany). The slices were then stained with 4% uranyl acetate (10 min) and 0.5% lead citrate (10 min) at room temperature. Finally, the ultrathin sections were visualized and photographed by using the TEM (Tecnai G2 Spirit, FEI, Holland).

### Statistical Analysis

Statistical evaluation of the data was performed using data statistics software GraphPad Prism 8 (GraphPad Software Inc., San Diego, CA, USA) and SPSS 24.0 software (SPSS, Inc., Chicago, IL, USA). All of the measurement data in this article were tested for normality first. Median (interquartile range) percentiles were used to express data that were not consistent with normal distribution, and Mann-Whitney *U*-tests were used to analyze the difference between groups. The normally distributed data were presented as the mean ± SD. The ANOVA followed by Tukey's *post-hoc* analysis was applied to analyze the difference between multiple groups. The value of *p* < 0.05 was considered as a significant difference.

## Results

### Animal Mortality and SAH Grade

**Figure 1:**
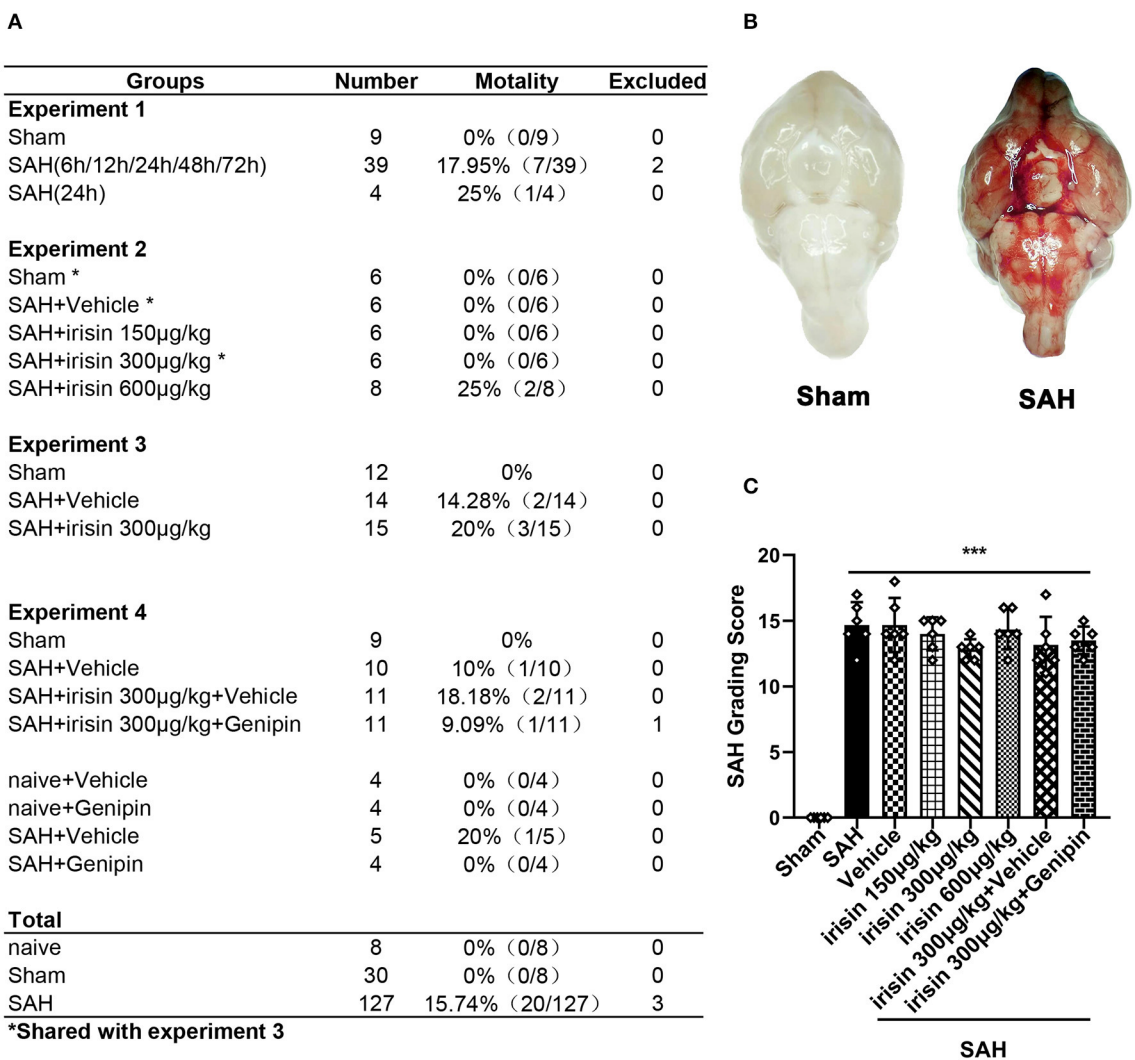
Animal usage and SAH grade. **(A)** The record of the animal used in this experiment. **(B)** Representative pictures of brains in the sham and SAH groups. **(C)** SAH grade scores in each group. Data were represented with mean ± SD, *n* = 6 per group; the one-way ANOVA was used followed by the Tukey's HSD *post-hoc* test and the Holm–Bonferroni correction method. ***P < 0.001 vs. sham group. Vehicle group, sterile 0.9% of NaCl; SAH, subarachnoid hemorrhage.

A total of 165 animals were used in this study, of which 8 were used in the naive group, 30 were used in the sham group, and 127 were subjected to induce the SAH model (Figure 1A). There was no mortality in the naive group and the sham group, while 20 (15.74%) mice died after SAH. Three mice were excluded from the study due to mild SAH (SAH grade < 8). No significant difference in SAH grades was observed among all SAH groups at 24 h after SAH (Figures 1B,C).

### Time Course and Spatial Change of Endogenous FNDC5/Irisin Levels in Ipsilateral Hemisphere at 24 h After SAH

**Figure 2:**
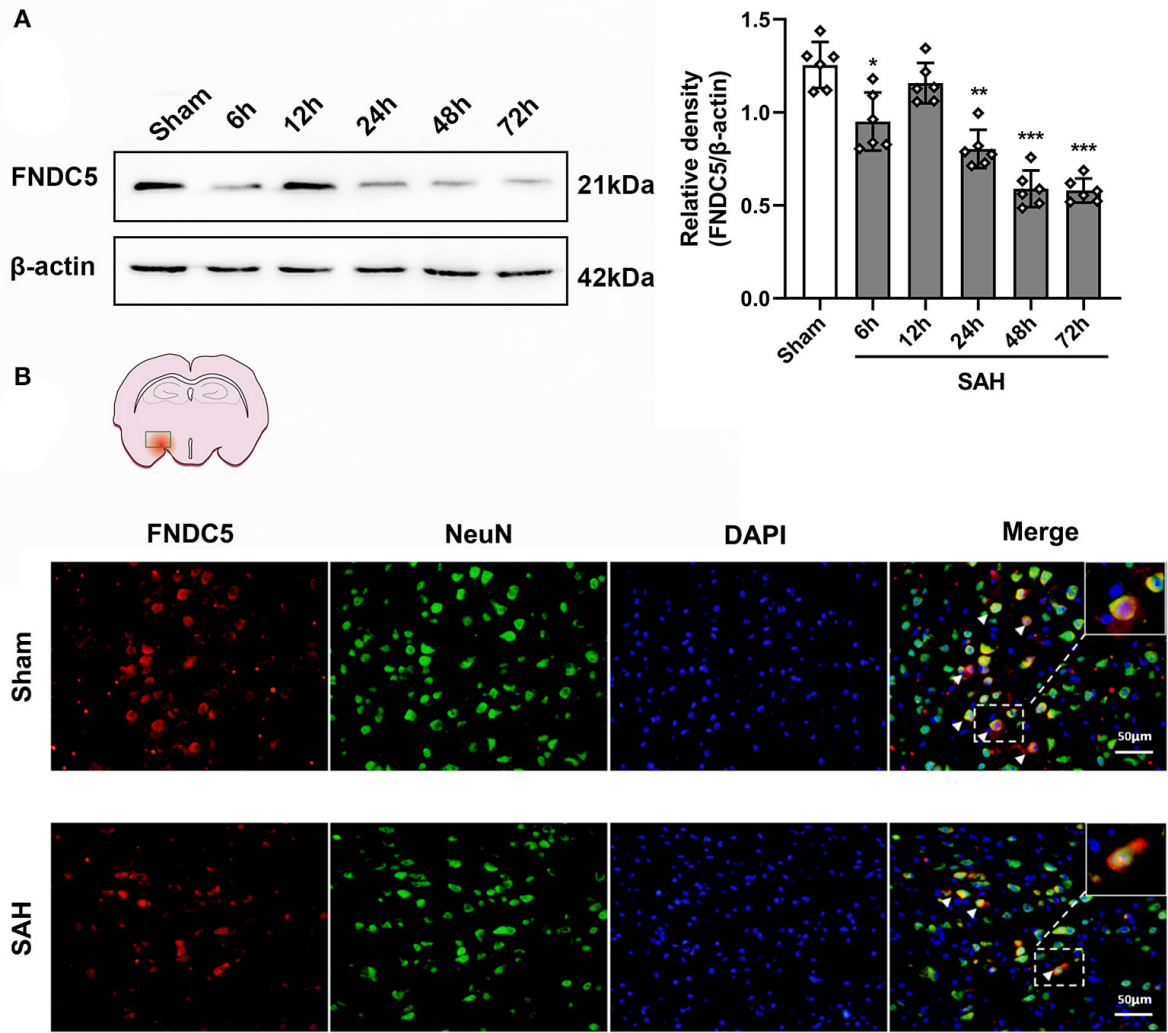
Time course of FNDC5/irisin expression after SAH. **(A)** Representative western blotting images and relative density analysis of FNDC5/irisin at 6, 12, 24, 48, and 72 h after SAH. Endogenous FNDC5/irisin protein decreased at 6 h while it increased at 12 h and decreased again from 24 to 72 h after SAH. Data were represented as mean ± SD, *n* = 6 per group; the one-way ANOVA was used followed by the Tukey's HSD *post-hoc* test and the Holm–Bonferroni correction method. *P < 0.05, **P < 0.01, ***P < 0.001 vs. sham group. **(B)** FNDC5/irisin could be co-localized with neurons, and the number of FNDC5/irisin-positive neurons was decreased at 24 h after SAH. Scale bar = 50 μm, n = 3 per group; FNDC5, fibronectin domain-containing protein 5; SAH, subarachnoid hemorrhage.

Western blotting results displayed that endogenous FNDC5/irisin protein decreased at 6 h after SAH and increased at 12 h and decreased again at 24 h after SAH when compared to the sham group (Figure 2A). Double immunofluorescence staining showed that FNDC5/irisin could be co-localized with neurons in the mouse brain. Meanwhile, the number of FNDC5/irisin-positive neurons decreased at 24 h after SAH when compared with that in the sham group (Figure 2B).

### Short-Term Neurological Functions Were Improved by Exogenous Irisin Treatments After SAH

**Figure 3:**
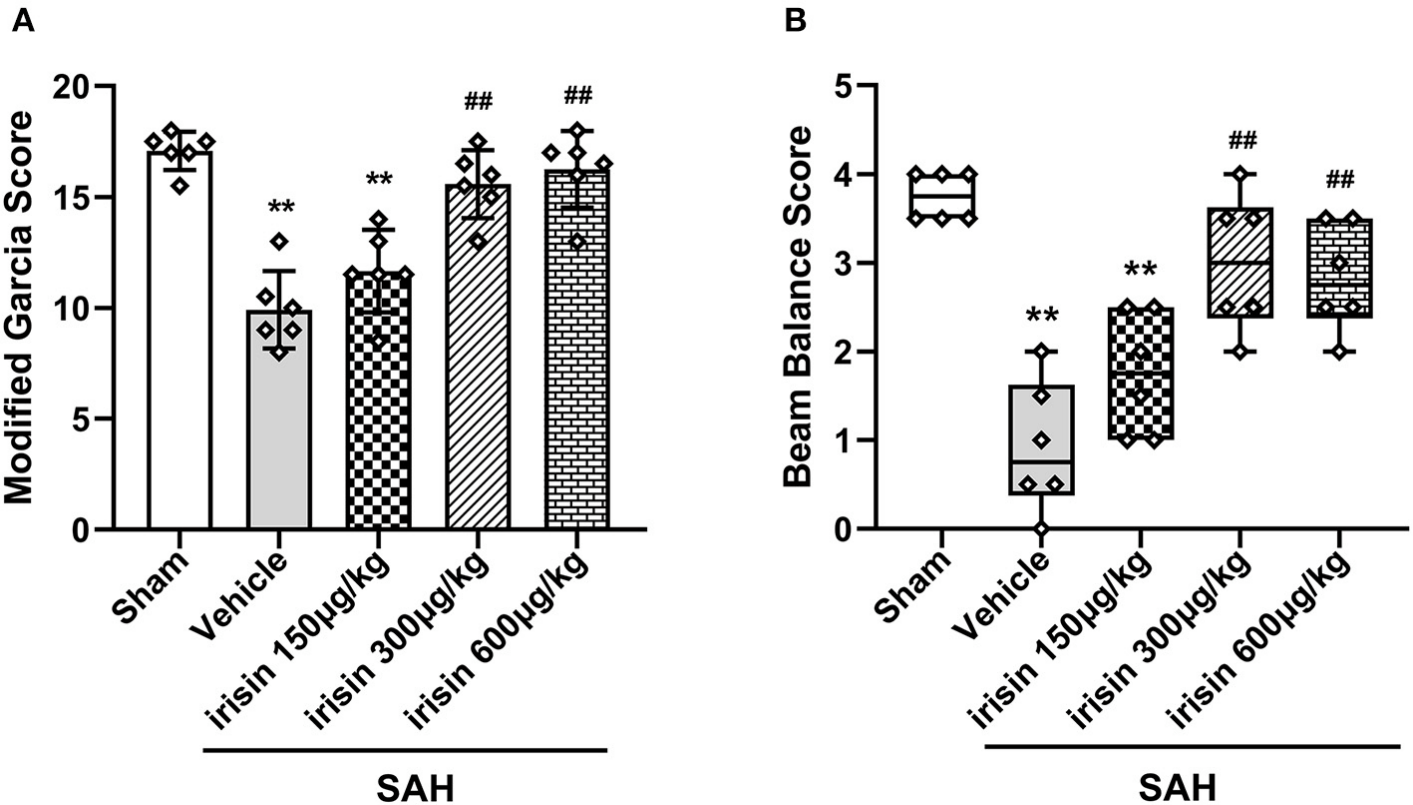
Beneficial effects of irisin on neuronal function 24 h after SAH. **(A,B)** Treatment with irisin significantly improved neurological deficits at 24 h after SAH and 300 μg/kg irisin was selected as the effective dose, *n* = 6 per group. The modified Garcia scores were represented as mean ± SD; the one-way ANOVA was used followed by the Tukey's HSD *post-hoc* test and the Holm–Bonferroni correction method. The beam balance test scores were represented as median 25–75th percentiles, and the Mann–Whitney *U*-test and the Kruskal–Wallis test followed by the Steel–Dwass test for multiple comparisons were used to analyze the difference between groups. **P < 0.01, ^##^*P* < 0.01 vs. SAH + Vehicle group. Vehicle group, sterile 0.9% of NaCl; SAH, subarachnoid hemorrhage.

At 24 h after SAH, worse neurobehavioral performances of animals in the vehicle group were observed in the modified Garcia scale and the beam balance test when compared to the sham group. Compared with animals in the vehicle group, exogenous irisin treatment at a middle dose (300 μg/kg) and a high dose (600 μg/kg) significantly improved the neurological performance of those animals at 24 h after SAH (Figures 3A,B). Based on the results, the dosage of 300 μg/kg was chosen for subsequent experiments.

### Neuronal Apoptosis Was Ameliorated by Exogenous Irisin Treatment After SAH

**Figure 4:**
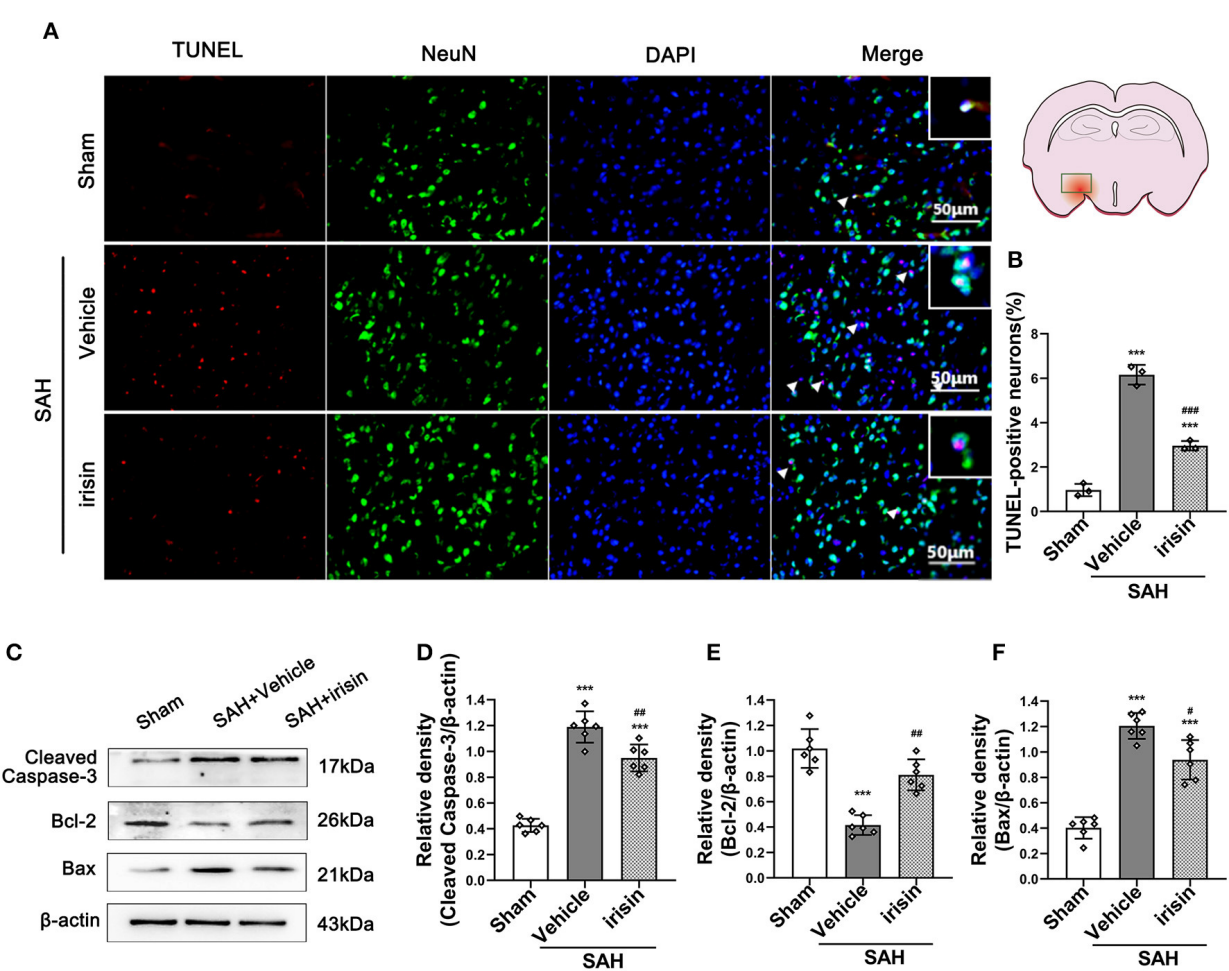
Anti-apoptosis effects of exogenous irisin treatment on neurons 24 h after SAH. **(A,B)** TUNEL-positive neurons were significantly decreased after exogenous irisin administration. Scale bar = 50 μm, *n* = 3 for each group. **(C–F)** Representative western blotting images and relative density analysis of Bax, Bcl-2, and cleaved caspase-3 at 24 h after SAH. Exogenous irisin treatment significantly improved the expressions of Bcl-2 **(E)** and result in a decreased level of apoptotic marker cleaved caspase-3 **(D)** and Bax **(F)**, *n* = 6 for each group. Data were presented as mean ± SD. The one-way ANOVA was used followed by the Tukey's HSD *post-hoc* test and the Holm–Bonferroni correction method. ***P < 0.001 vs. Sham group; ^#^*P* < 0.05, ^##^*P* < 0.01, ^###^*P* < 0.001 vs. SAH+Vehicle group. Vehicle group, sterile 0.9% of NaCl; TUNEL, TdT-UTP nick end labeling.

The pathological change of neuronal apoptosis was caused by SAH stroke. Co-staining of neurons and TUNEL-positive cells were performed to detect the effects of exogenous irisin. Evident neuronal apoptosis was observed in the vehicle group after SAH, while the number of TUNEL-positive neurons decreased after irisin administration (Figures 4A,B). Western blotting was used to semi-quantify the protein levels related to apoptosis (Figures 4C–F). By the evident elevation of the cleaved caspase-3 protein level, apoptosis in the brain tissue was confirmed (Figures 4C,D). Irisin treatment alleviated this injury, for decreasing the protein level of the cleaved caspase-3, when compared to the vehicle group. Bax and Bcl-2, the pro-apoptotic marker and the anti-apoptotic marker, were also assessed to confirm these results. Evidently, the level of the Bax protein was increased and the Bcl-2 protein was decreased after SAH, when compared to the sham group. However, irisin treatment reversed these changes. Immunoblot data showed that the Bax protein decreased in the irisin-treated group while the Bcl-2 protein increased in the irisin-treated group, when compared to the vehicle group (Figures 4C,E,F).

### Oxidative Stress Insults Were Alleviated by Exogenous Irisin Treatment After SAH

**Figure 5:**
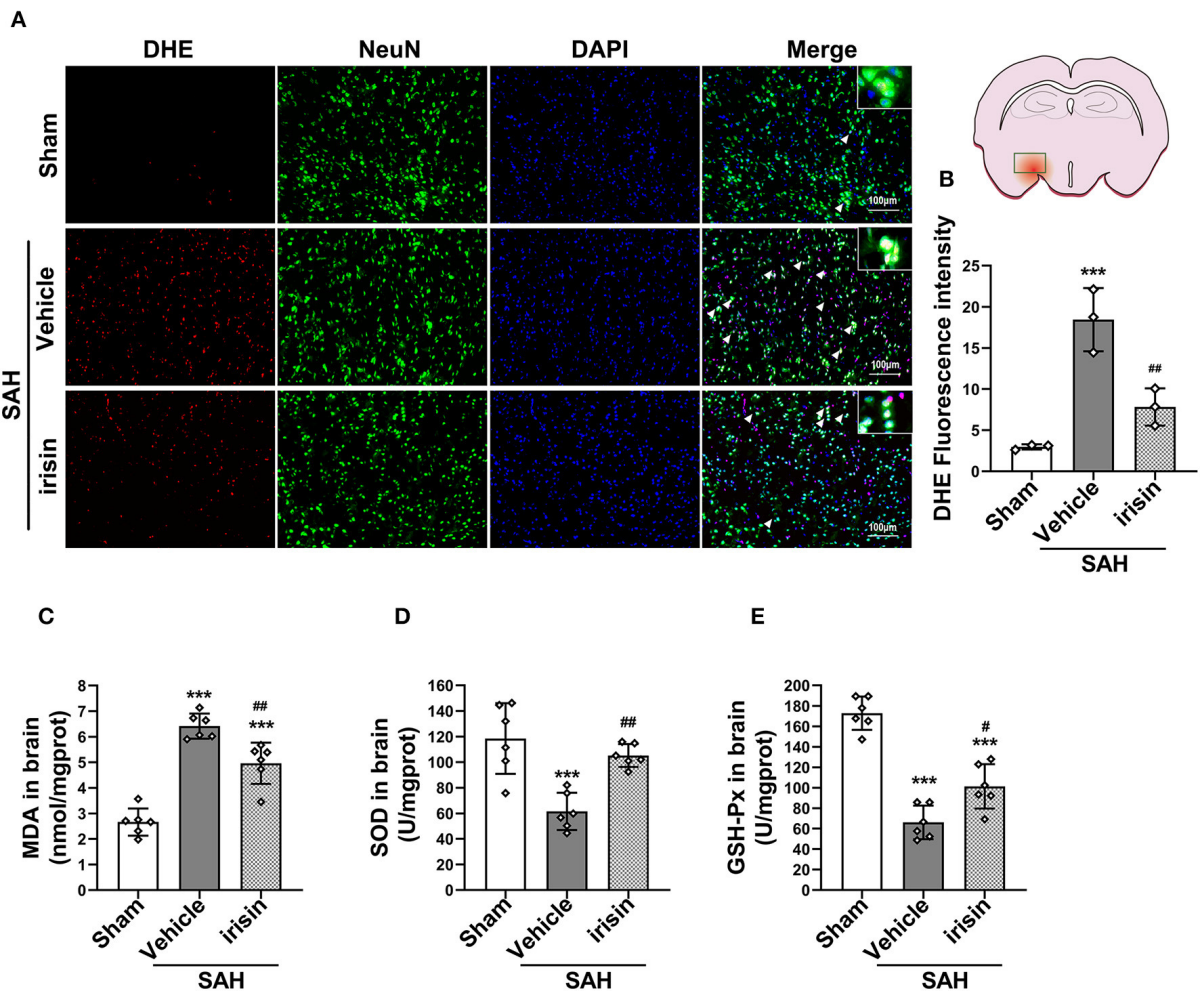
Effect of exogenous irisin treatment on SAH-induced oxidative stress 24 h after SAH. **(A,B)** A lower red fluorescence intensity (DHE) was observed in the irisin-intervened group, Scale bar = 100 μm, *n* = 3 for each group. **(C)** Levels of oxidative markers were analyzed, MDA levels were significantly decreased after irisin treatment. **(D,E)** SOD and GSH-PX levels were increased with the irisin intervention, *n* = 6 for each group. Data were presented as mean ± SD. The one-way ANOVA was used followed by the Tukey's HSD *post-hoc* test and the Holm–Bonferroni correction method. ***P < 0.001 vs. Sham group; ^#^*P* < 0.05, ^##^*P* < 0.01 vs. SAH + Vehicle group. Vehicle, sterile 0.9% of NaCl; DHE, dihydroethidium; SOD, Superoxide Dismutase; GSH-PX, Glutathione Peroxidase Activity.

After SAH stroke, the oxidative stress reaction was presented in the cascade insults. ROS, as a crucial factor in the oxidative stress reaction, was evaluated by DHE staining (Figures 5A,B). Our data revealed a significantly high red fluorescence intensity in the vehicle group when compared to the sham group. However, irisin administration ameliorated the oxidative stress insult, as lower red fluorescence intensity and lower number of DHE-positive cells were observed in the irisin-intervened group than those in the vehicle group. Other markers of oxidative stress, involving MDA, SOD, and GSH-PX, were examined to confirm the above results (Figures 5C–E). As our data showed, the activity of SOD and GSH-PX decreased after irisin treatment, while the level of MDA was increased, when compared with the vehicle group.

### The Mitochondrial Morphology and Mitochondrial Biogenesis Were Maintained by Exogenous Irisin Treatment After SAH

**Figure 6:**
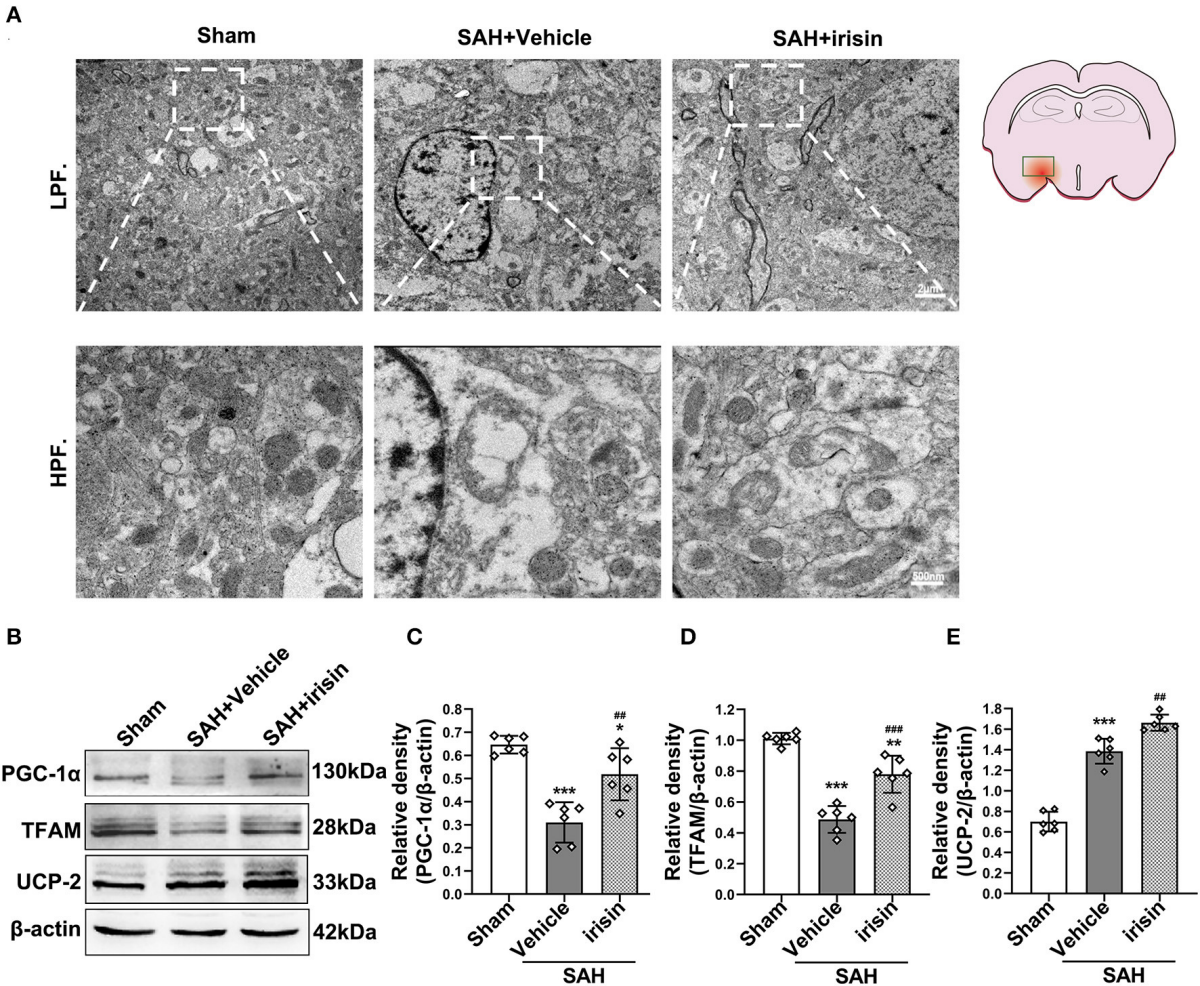
Effect of exogenous irisin treatment on mitochondrial morphology and mitochondrial biogenesis in mice brain 24 h after SAH. **(A)** Less mitochondrial swelling and vacuolization were observed under the TEM after irisin treatment, Scale bar = 2 μm, 500 nm, *n* = 3 for each group. **(B–E)** Representative western blotting images and relative density analysis of TFAM, PGC-1α, and UCP-2 24 h after SAH. Exogenous irisin treatment significantly improved the expressions of TFAM and PGC-1α. The UCP-2 expression increased after SAH and a higher level was observed after irisin treatment, *n* = 6 for each group. All data were presented as mean ± SD. The one-way ANOVA was used followed by the Tukey's HSD *post-hoc* test and the Holm–Bonferroni correction method. *P < 0.05, **P < 0.01, ***P < 0.001 vs. Sham group; ^##^*P* < 0.01, ^###^*P* < 0.001 vs. SAH + Vehicle group. Vehicle group, sterile 0.9% of NaCl; UCP-2, Uncoupling Protein-2; SAH, subarachnoid hemorrhage.

Transmission electron microscopy was conducted to observe the mitochondrial dysfunction after SAH around hemorrhage regions. Images captured by TEM suggested the imbalance of mitochondrial dynamics, which is the initial process of cell apoptosis after SAH. In the irisin-treated group, these changes were relieved, and the number of swelling mitochondria and vacuoles were decreased (Figure 6A). Mitochondrial biogenesis is essential for maintaining normal mitochondrial function. Markedly, this kind of self-regulation was disturbed after SAH. TFAM and PGC-1α, two key proteins related to mitochondrial biogenesis, were significantly decreased after SAH cascade insults when compared with those in the sham group. However, there was an evident upregulation of these two proteins observed after the treatment of irisin (Figures 6B–D). Furthermore, although the SAH stroke may stimulate the UCP-2 increase by a feedback regulation mechanism, the irisin treatment enhanced this promotion of UCP-2 in the SAH + irisin group (Figures 6B,E).

### The Neuroprotective Effects of Irisin Were Reversed by the Inhibition of UCP-2

**Figure 7:**
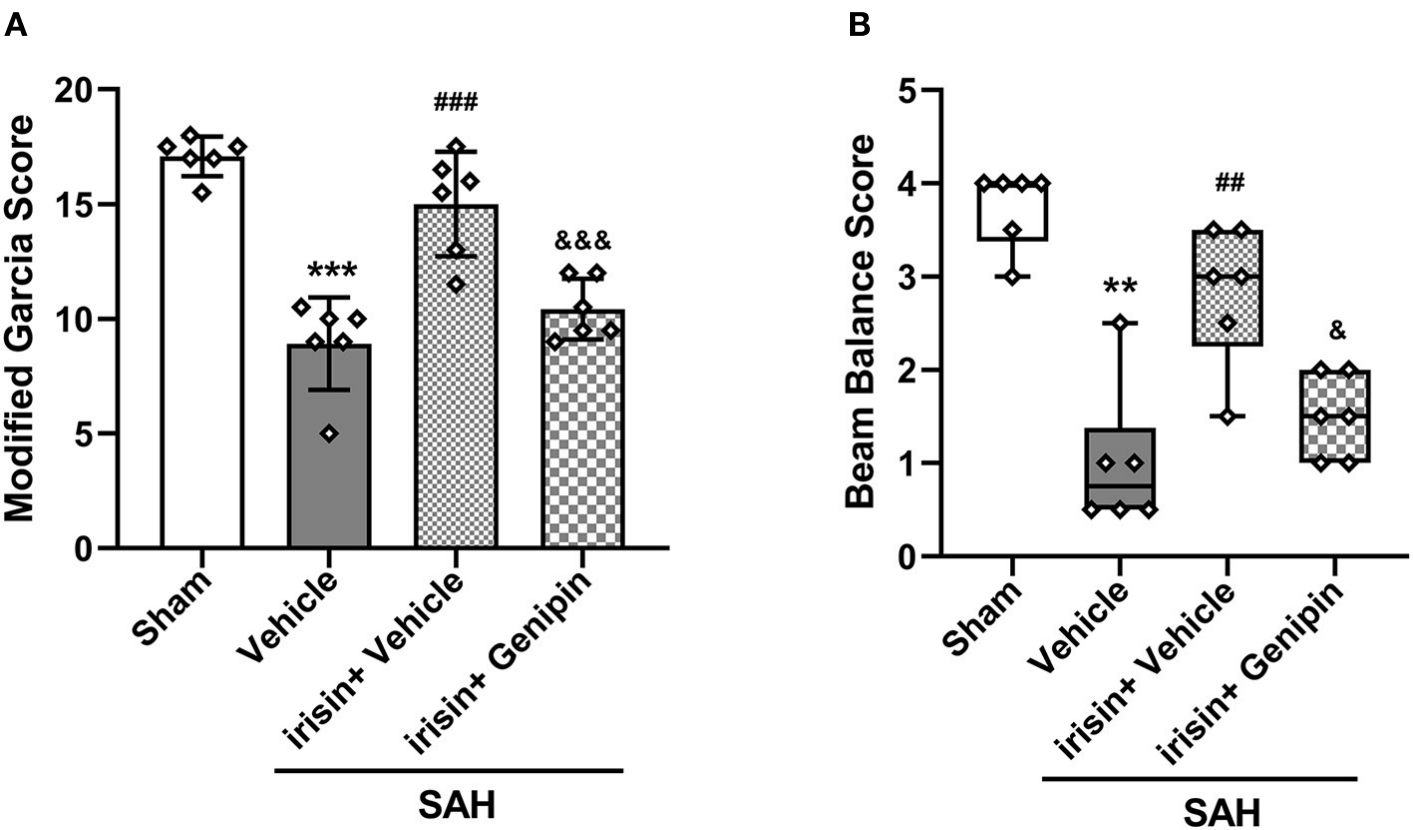
Inhibition of UCP-2 pathway blunted the beneficial effect of irisin treatment on short-term neurologic function after SAH. **(A,B)** Genipin significantly blunted the protective effect of irisin treatment on neurological deficits at 24 h after SAH, *n* = 6 for each group. The modified Garcia test scores were represented as mean ± SD; the one-way ANOVA was used followed by the Tukey's HSD *post-hoc* test and the Holm–Bonferroni correction method. The beam balance test scores were represented as median 25th−75th percentiles, and the Mann–Whitney *U*-test and the Kruskal–Wallis test followed by the Steel–Dwass method of multiple comparisons were used to analyze the difference between groups. **P < 0.01, ***P < 0.001 vs. Sham group; ^##^*P* < 0.01, ^###^*P* < 0.001 vs. SAH + Vehicle group; ^&^*P* < 0.05, ^&&&^*P* < 0.001 vs. SAH + irisin + Vehicle group. Vehicle group, sterile 0.9% of NaCl; SAH, subarachnoid hemorrhage.

**Figure 8:**
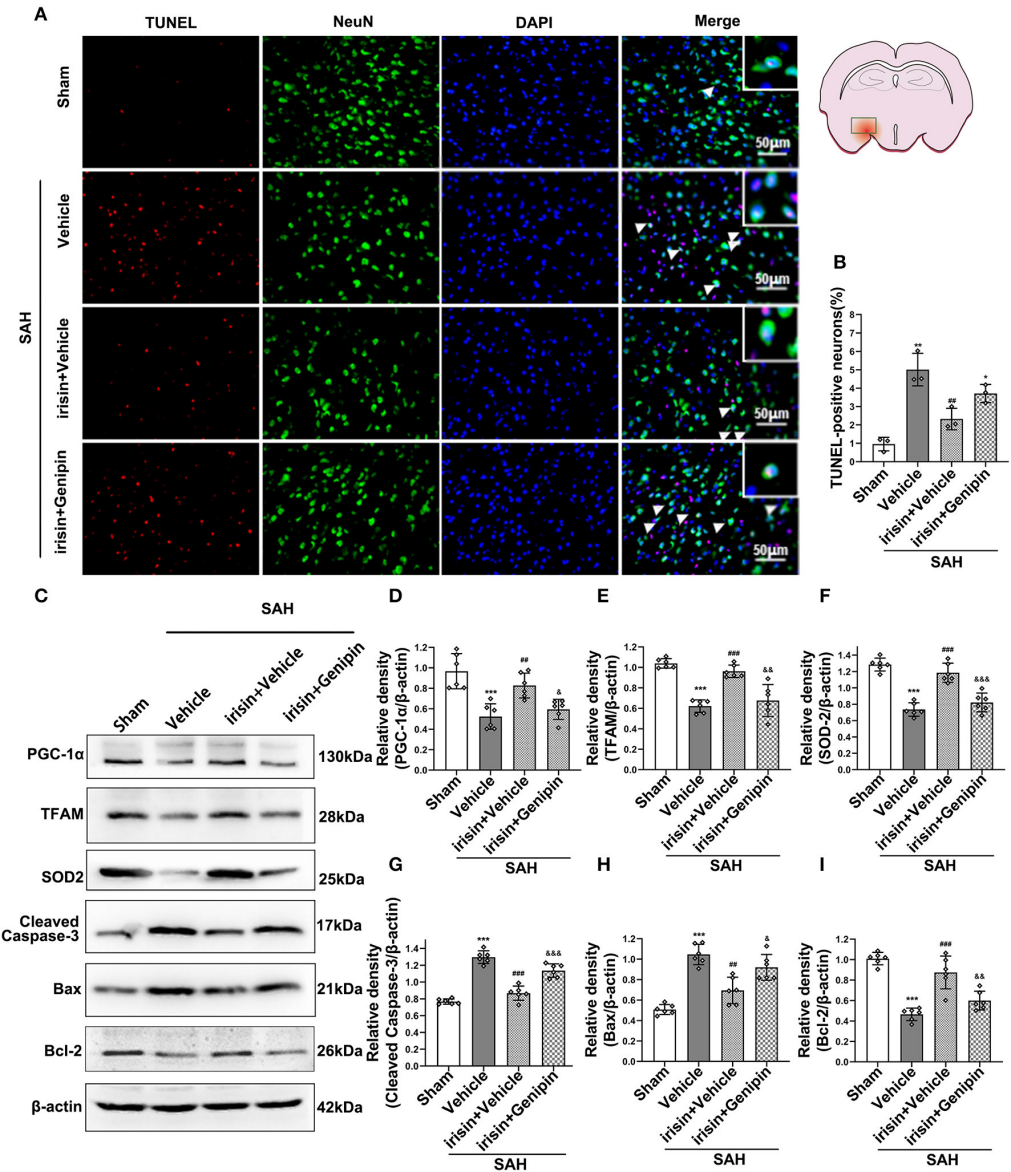
Inhibition of UCP-2 pathway reversed the beneficial effects of irisin on apoptosis, mitochondrial function, and oxidative stress after SAH. **(A,B)** TUNEL-positive neurons were significantly increased after genipin administration in the irisin treatment group. Scale bar = 50 μm, *n* = 3 for each group (The area of observation was circled in red). **(C–I)** Representative western blotting images and relative density analysis of proteins related to mitochondrial biogenesis (TFAM, PGC-1α), oxidative stress (SOD2), and apoptotic markers (Cleaved caspase-3, Bax, Bcl-2) at 24 h after SAH. As an exogenous UCP-2 inhibitor, genipin significantly blunted the expressions of PGC-1α, TFAM, SOD2, and Bcl-2 **(D,E,F,I)** and resulted in an increased level of cleaved caspase-3 and Bax **(G,H)**, *n* = 6 for each group. All data were presented as mean ± SD. The one-way ANOVA was used followed by the Tukey's HSD *post-hoc* test and the Holm–Bonferroni correction method. *P < 0.05, **P < 0.01, ***P < 0.001 vs. Sham group; ^##^*P* < 0.01, ^###^*P* < 0.001 vs. SAH + Vehicle group. ^&^*P* < 0.05, ^&&^*P* < 0.01, ^&&&^*P* < 0.001 vs. SAH + irisin + Vehicle group. Vehicle group, sterile 0.9% of NaCl; TUNEL, TdT-UTP nick end labeling; SOD, Superoxide Dismutase; UCP-2, Uncoupling Protein-2.

Our data showed that genipin significantly reversed the neuroprotective effects of irisin after SAH. Firstly, in the preliminary experiment, genipin was confirmed to have no effect on neurological scores (Supplementary Figures 1A,B). Then, lower neurological scores of the modified Garcia scale and the beam balance test in the SAH+irisin+genipin group were obtained when compared to the SAH+irisin+vehicle group (Figures 7A,B). Similarly, co-immunofluorescence staining of neurons and apoptosis cells were applied to explore the relationship between the effects of irisin and the UCP-2 antagonist, genipin. A higher number of TUNEL-positive neurons were calculated in the SAH+irisin+Genipin group when compared to the SAH+irisin+vehicle group (Figures 8A,B). Semi-quantitative methods of proteins reconfirmed these results. Levels of apoptotic-related protein cleaved caspase-3, Bcl-2 and Bax were changed in the SAH+irisin+vehicle group as previously described when compared to the vehicle group. However, the application of genipin reversed the protective change (Figures 8C,G–I). Meanwhile, SOD2, which is an important component of antioxidant enzymes in biological systems, was decreased after the administration of genipin in the SAH+irisin+Genipin group when compared to the SAH+irisin+vehicle group (Figures 8C,F). Furthermore, we observed that, in the SAH+irisin+genipin group, the mitochondrial biogenesis markers, including TFAM and PGC-1α, were partially decreased after the administration of genipin, when compared to the SAH+irisin+vehicle group (Figures 8C–E).

## Discussion

**Figure 9:**
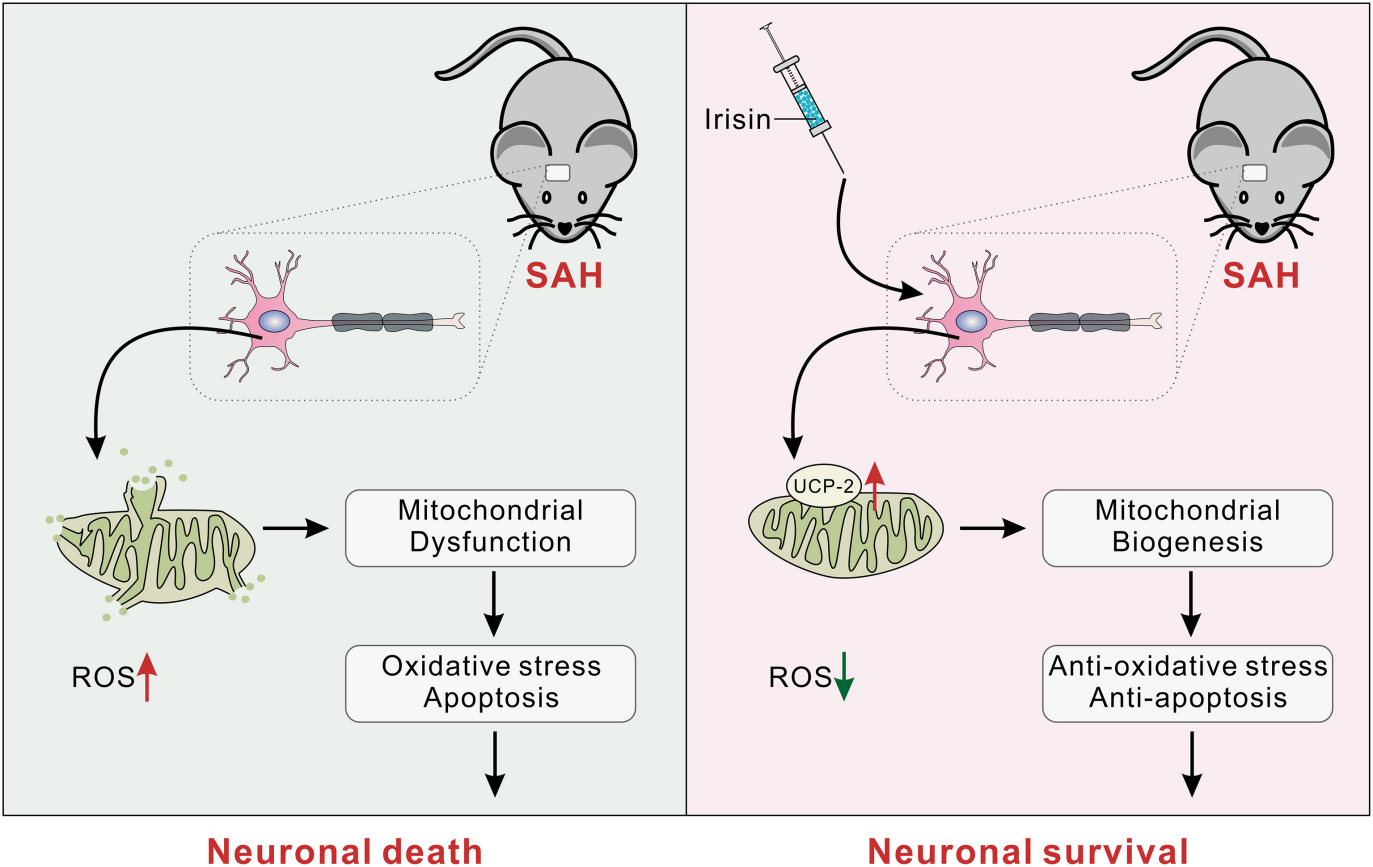
The schematic of irisin on mitochondrial biogenesis following SAH. SAH, subarachnoid hemorrhage.

Several novel findings were made in the present study: (1) endogenous irisin in the brain increased at 12 h while it decreased at 24 h after SAH; (2) intracerebroventricular administration of recombinant irisin significantly attenuated neurobehavioral deficits of the experimental mice at 24 h after SAH; (3) neuronal apoptosis and excessive oxidative stress, which were key pathologic processes in the process of EBI, could be alleviated by irisin administration; (4) the potential mechanism of the neuroprotective effects of irisin might be related to the maintenance of mitochondrial homeostasis; and (5) the inhibitor of UCP-2 partially offset the beneficial effects of exogenous irisin. Thus, irisin may regulate neuronal apoptosis and excessive oxidative stress, at least partially, through the UCP-2 pathway (Figure 9).

In the present study, we found that the protein level of irisin in the ipsilateral hemisphere increased at 12 h while it decreased at 24 h after injury. Interestingly, currently available evidence could not explain how this brain injury affects the expression of FNDC5 and the secretion of irisin. We found several possibilities. Firstly, in most instances, the hormones would be redistributed by the self-regulating system of the body to respond to emergencies. Thus, the irisin level increased in the compensatory period. However, at 24 h after SAH, which was reported to be the key time-point of the EBI phase, the oxidative stress and the subsequent cascade response may disturb the self-regulation and reduce the irisin levels. Secondly, the normal movement of the post-SAH mice would be reduced, thus the reduced muscle metabolism would be another reason for the downregulation of the exercise-liked irisin. Finally, the acute stroke event would affect the neuron-bone-muscle axon, which might also attenuate irisin levels after SAH.

Previous researches have gradually demonstrated the neuroprotective roles of irisin (Li et al., 2017; Asadi et al., 2018; Tu et al., 2018; Lourenco et al., 2019; Wu H. et al., 2019). In the experimental models of neurodegenerative disorders, irisin showed remarkable abilities in memory enhancing and synaptic remodeling (Kim and Song, 2018; Lourenco et al., 2019). Meanwhile, in brain injury models, the beneficial roles of irisin are thought to be exerted against the deterioration of these cascade lesions in EBI, especially about the apoptosis and the oxidative stress (Li et al., 2017; Guo et al., 2019). For example, Guo et al. (2019) suggested that irisin peptide could attenuate brain damage both morphologically and functionally, as well as protect the blood–brain barrier (BBB) from disruption after focal cerebral ischemia/reperfusion. In line with this, Li et al. (2017) demonstrated that, by activating the Akt and ERK1/2 signaling pathways, irisin can reduce ischemia-induced neuronal injury. After brain ischemic stroke, exogenous irisin treatment inhibited the activation of Iba-1^+^ and MPO-1^+^ cells, which are the markers of microglia and neutrophils. Meanwhile, the expression of pro-inflammatory cytokines mRNA as IL-6 and TNF-α were reduced. More importantly, the levels of the oxidative stress parameters, involving nitrotyrosine (NO2-Tyr), superoxide anion, 4-hydroxynonenal (4-HNE), were also decreased after irisin administration. This evidence indicated the potential anti-inflammatory and antioxidant properties of irisin. In clinical research, irisin was also reported as a prognostic indicator in patients after ischemic stroke (Tu et al., 2018; Wu H. et al., 2019).

Neuronal apoptosis, which directly leads to neuronal death and function loss, was thought to be one of the main reasons for neurological deficits (Pang et al., 2018). Accumulative evidence displayed that irisin can play an anti-apoptotic role in multiple disease models (Natalicchio et al., 2017; Askari et al., 2018; Pang et al., 2018; Storlino et al., 2020). Even in the brain stroke models, researchers have validated the property of irisin. As Asadi et al. (2018) reported, irisin peptide could protect brain tissues against ischemic damage by reducing neural apoptosis. Meanwhile, irisin administration reduced brain edema without disturbing BBB permeability. In the present study, TUNEL-positive cells were decreased after irisin administration. Also, the pro-apoptotic marker Bax and caspase-3 activation were significantly reduced, while the anti-apoptotic marker Bcl-2 was increased in the irisin-treated group after SAH. These results revealed irisin represented an anti-apoptotic effect after SAH.

Notably, excessive oxidative stress could also trigger neuronal apoptosis, which is one of the common characteristics of EBI (Mo et al., 2019; Pang et al., 2019). As our previous study expounded, strategies to block oxidative stress-related pathways could improve the outcomes after SAH (Pang et al., 2018). The ROS, which would be excessively produced after acute brain injury, was recognized as the crucial factor in the oxidative stress reaction (Fan et al., 2017). When the endogenous anti-oxidant system could not effectively scavenge ROS, oxidative stress was aggravated, followed by cell death (Le Belle et al., 2011). Consistent with our findings, irisin has been shown to have a latent anti-oxidative property (Li et al., 2017; Askari et al., 2018; Bi et al., 2019). Our data showed that irisin administration could reduce oxidative stress as measured by the expression of anti-ROS markers, SOD and GSH-PX, and the pro-ROS marker, MDA, as well as DHE staining. It is not clear, however, how irisin mediates these interlaced pathological processes after SAH.

Cerebral metabolism is highly active, and the maintenance of these metabolic activities depends on the energy provided by mitochondria. After brain damage, the energy engine, mitochondria, could not meet the urgently needed energy demand (Sheng and Cai, 2012). Then, the imbalance of mitochondrial dynamics would be the initial process of numerous deleterious cascades, including inflammatory infiltration, cell apoptosis, excessive ROS generation, and oxidative stress damage (Sims and Muyderman, 2010). These pathological processes commonly develop in the EBI process as previously mentioned in Mo et al. (2019). However, to maintain homeostasis, mitochondria are continually undergoing division and fusion (Fan et al., 2017). More importantly, mitochondrial biogenesis is also happening, which is one of the main ways to supplement the nascent mitochondria (Pfanner et al., 2019).

As previously reported, mitochondrial morphology is vital for sustaining mitochondrial dynamics after cerebral injury (Wei et al., 2019; Lai et al., 2020). In our current study, the results observed from TEM indicated that nascent mitochondria were increased, with less vacuole and less swelling mitochondria after irisin treatment. The mitochondrial transcription factor A (TFAM), was also evaluated to be increased by the irisin treatment after SAH. In parallel, the increased expression of PGC-1α was observed in the irisin treatment group. Previous studies revealed that in the regulation of the mitochondrial biological activities, especially for mitochondrial biogenesis, PGC-1α played a crucial role (Fernandez-Marcos and Auwerx, 2011). Recent literature also confirmed that the upregulation of neuronal PGC-1 α, the nodal regulator of mitochondrial biogenesis, would help to ameliorate cognitive impairment induced by chronic cerebral hypoperfusion (Han et al., 2020). Thus, these results indicated that irisin may be performing the roles of maintaining mitochondria morphology, as well as promoting mitochondrial biogenesis to complement the normal mitochondria after SAH. Since the normal mitochondrial functions were maintained, subsequent cascades, including apoptosis and oxidative stress, would be alleviated.

Numerous molecular regulatory processes might be related to mitochondrial biogenesis (Pfanner et al., 2019). It is noteworthy that UCP-2, an endogenous inducible protein located in the inner membrane of mitochondria, is thought to be one of the protagonists in the UCP family for regulating mitochondrial metabolism and ROS generation (Mattiasson et al., 2003; Mattiasson and Sullivan, 2006; Mailloux and Harper, 2011). By promoting mitochondrial biogenesis, UCP-2 can directly regulate synaptic plasticity and neurotransmission, which are important in supporting neuron function and survival (Pfanner et al., 2019). Notably, the UCP-2 protein level increased after the SAH in our experiment, which is in line with a previous study (Mo et al., 2019). Early brain damage could lead to excessive ROS production, subsequently, activating UCP-2 to induce proton leak (Mailloux and Harper, 2011; Mo et al., 2019). After brain injury, the UCP-2 promotion could be a self-protective regulatory mechanism, for it could provide negative feedback for ROS production (Mailloux and Harper, 2011). Strikingly, after the irisin treatment, the level of UCP-2 protein was further increased, which was in parallel with the increase of the mitochondrial biogenesis marker proteins. Consistent with our conjecture, the inhibition of UCP-2 by genipin decreased the PGC-1α and TFAM levels, indicating that the mitochondrial biogenesis process would be impacted partially. In agreement with these observations, we further found that the inhibition of UCP-2 significantly reversed the anti-apoptosis and anti-oxidative stress effects of irisin. These results revealed an important functional loop between mitochondrial biogenesis and irisin, which might be, at least in part, regulated by UCP-2 protein.

It is noteworthy that exercise is closely related to the level of increase of the endogenous irisin. Meanwhile, irisin is determined as one of the endogenous molecules to exert neuroprotective effects (Li et al., 2017; Lourenco et al., 2019). For those suffering from acute or chronic nervous system disorders, physical exercise is well-accepted as an effective approach for reducing the risk of illness and improving the outcomes of patients (Dobkin, 2008; Tan et al., 2014). On the other hand, the rupture of intracranial aneurysm is one of the main incentives of SAH; thus, the patients who bear intracranial aneurysm would be advised not to do strenuous exercise; otherwise, it could cause a ruptured aneurysm. Our research might help to establish alternative approaches for those patients to share the exercise benefits, while more clinical data are needed to support the notion.

There are still several limitations to our study. Firstly, in the system of skeletal and muscle, the receptor (s) of irisin has been gradually excavated (Kim et al., 2018; Farmer, 2019). However, the irisin related receptor (s) and targets in CNS are still needed to be identified in future studies. Second, this study focused on irisin-mediated mitochondrial biogenesis and UCP-2 related targets. We could not exclude that the neuroprotective effects of irisin were the results of other forms of mitochondria protections. Third, although the intracerebroventricular administration of irisin assured the protein could perform its roles in CNS, a cross-talk of the influence between the irisin serum concentration and cerebrospinal fluid (CSF) should be illustrated more clearly. The relevance of actual irisin plasma levels with or without exercise would be researched in future studies. Last, considering the numerable variables of the female rodents and the influence of estrogen, we only used male mice for this study. The results would be more convincing if the experiment could be carried out in both female and male mice.

## Conclusion

The current study suggests that irisin exerts anti-apoptosis and anti-oxidative stress roles in the process of the EBI phase after experimental SAH, potentially by maintaining mitochondrial homeostasis. Focusing on metabolic-related cytokine and targeting mitochondria-centered pathological cascades, a novel therapeutic strategy to address the challenges in EBI is emerging.

## Data Availability Statement

The original contributions presented in the study are included in the article/Supplementary Material, further inquiries can be directed to the corresponding author/s.

## Ethics Statement

The animal study was reviewed and approved by Animal Committee of the Ethics Committee of Southwest Medical University.

## Author Contributions

TT designed and performed the experiment, collected and analyzed the data, and prepared the manuscript. SY and JPa were involved in the experiment design. XZ, LZ, and YZ were involved in behavioral testing and biochemical analysis. YX and KG were involved in preparing the animal models and participated in interpreting the results. LC participated in the manuscript revision. JPe and YJ contributed to the study concept and design, secured funding for the project, and prepared and critically revised the manuscript. All authors contributed to the article and approved the submitted version.

## Conflict of Interest

The authors declare that the research was conducted in the absence of any commercial or financial relationships that could be construed as a potential conflict of interest.
