# Supplementary material for: Irisin Contributes to Neuroprotection by Promoting Mitochondrial Biogenesis After Experimental Subarachnoid Hemorrhage
Source: Front Aging Neurosci. 2021 Feb 3;13:640215. doi: 10.3389/fnagi.2021.640215 (PMC7886674; doi:10.3389/fnagi.2021.640215)
Supplement: Supplementary file 1 [file Table_1.DOCX]

**Irisin contributes to neuroprotection by promoting mitochondrial biogenesis after experimental subarachnoid hemorrhage**

**Tianqi Tu^1^, Shigang Yin^2,3^, Jinwei Pang^1,3,4^, Xianhui Zhang^4^, Lifang Zhang^4^, Yuxuan Zhang^1^, Yuke Xie^2^, Kecheng Guo^2^, Ligang Chen^1,3,4^, Jianhua Peng^1,2,4*^, Yong Jiang^1,2,3,4*^**

^1^Department of Neurosurgery, the Affiliated Hospital of Southwest Medical University, Luzhou 646000, China.

^2^Laboratory of Neurological Diseases and Brain Function, the Affiliated Hospital of Southwest Medical University, Luzhou 646000, China.

^3^Academician (Expert) Workstation of Sichuan Province, the Affiliated Hospital of Southwest Medical University, Luzhou 646000, China.

^4^Sichuan Clinical Research Center for Neurosurgery, the Affiliated Hospital of Southwest Medical University, Luzhou 646000, China.

*** Correspondence:**Yong Jiang, PhD
[jiangyong@swmu.edu.cn](mailto:jiangyong@swmu.edu.cn)

Jianhua Peng, MD
[pengjianhua@swmu.edu.cn](mailto:pengjianhua@swmu.edu.cn)

**Supplementary Fig. S1**


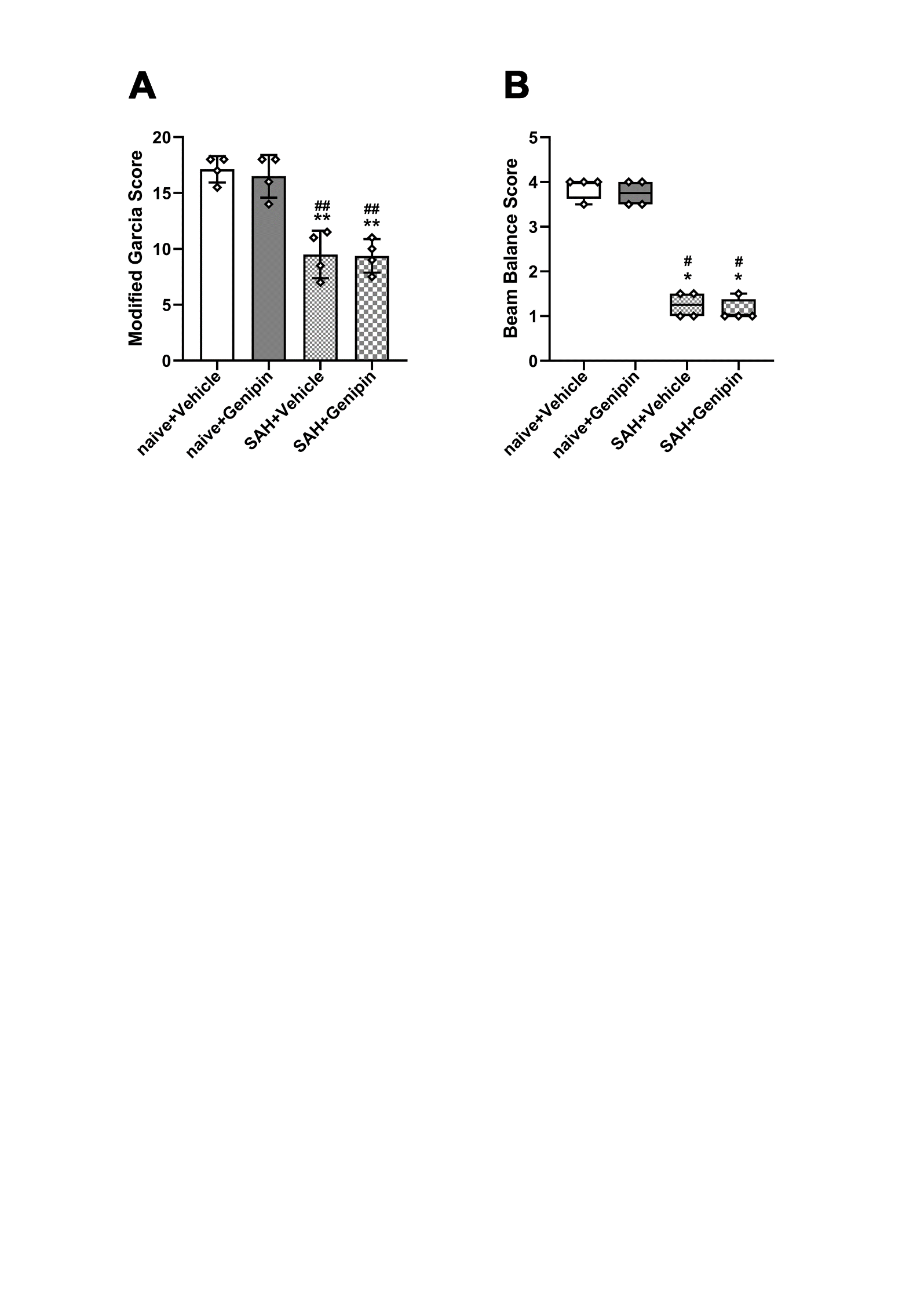


**Supplementary Fig. S1 Inhibition of UCP-2 pathway had no obvious effect on short-term neurologic function of each group. (A, B)** Genipin administration do not have significant effect on neurological scores of each group, n = 4 for each group. Modified Garcia scores were represented as mean ± SD, one-way ANOVA was used followed by Tukey's HSD post hoc test and Holm-Bonferroni correction. Beam balance scores were represented as median 25th-75th percentiles, and Mann-Whitney U-tests and Kruskal-Wallis tests followed by Steel-Dwass multiple comparisons were used to analyze the difference between groups. ^*^*P* < 0.05, ^**^*P* < 0.01 vs. naïve + Vehicle group; ^#^*P* < 0.05, ^##^*P* < 0.01 vs. naïve + Genipin group. Vehicle, sterile 0.9% of NaCl.
